# JMJD3 suppresses tumor progression in oral tongue squamous cell carcinoma patients receiving surgical resection

**DOI:** 10.7717/peerj.13759

**Published:** 2022-07-13

**Authors:** Yen-Hao Chen, Chang-Han Chen, Chih-Yen Chien, Yan-Ye Su, Sheng-Dean Luo, Shau-Hsuan Li

**Affiliations:** 1 Division of Hematology-Oncology, Department of Internal Medicine, Kaohsiung Chang Gung Memorial Hospital, Kaohsiung, Taiwan; 2 School of Medicine, College of Medicine, Chang Gung University, Taoyuan, Taiwan; 3 Department of Nursing, School of Nursing, Fooyin University, Kaohsiung, Taiwan; 4 Institute of Medicine, Chung Shan Medical University, Taichung, Taiwan; 5 Department of Medical Research, Chung Shan Medical University Hospital, Taichung, Taiwan; 6 Department of Otolaryngology, Kaohsiung Chang Gung Memorial Hospital, Kaohsiung, Taiwan

**Keywords:** JMJD3, Oral tongue cancer, Squamous cell carcinoma, Surgery

## Abstract

**Background:**

Jumonji domain-containing-3 (JMJD3) is reported to be a histone H3 lysine 27 (H3K27) demethylase and a tumor suppressor gene. The present study designed to investigate the crucial role of JMJD3 in oral tongue squamous cell carcinoma (OTSCC) patients who received surgical resection.

**Methods:**

We enrolled a total of 156 OTSCC patients receiving surgical resection, including 73 patients (47%) with high expression of JMJD3 and 83 patients (53%) harboring low expression of JMJD3. Two OTSCC cell lines, SAS and Cal 27, were used to explore the modulation of cancer. GSK-J4, a potent inhibitor of JMJD3, was used to treat the two OTSCC cell lines. The Chi-square test was performed to examine between-group differences in categorical variables; the Kaplan–Meier method was used to investigate survival outcome in univariate analysis, and the Cox regression model was used for multivariate analysis.

**Results:**

The median follow-up period was 59.2 months and he five-year disease-free survival (DFS) and overall survival (OS) rates were 46.2% and 50.0%, respectively. Better five-year DFS (59% versus 35%) and five-year OS (63% versus 39%) were mentioned in patients with high expression of JMJD3 compared to those with low expression of JMJD3. High expression of JMJD3 was significantly associated with superior DFS and OS in the univariate and multivariate analyses. Following successful inhibition of JMJD3 by GSK-J4, western blotting analysis showed the decreased expression of Rb and p21.

**Conclusion:**

Our study showed that high expression of JMJD3 is a good prognostic factor in OTSCC patients who underwent surgical resection.

## Introduction

Head and neck squamous cell carcinoma (HNSCC) is one of the most aggressive malignancies and ranks the fifth leading cause of cancer mortality (Ministry of Health and Welfare of Taiwan, 2015). The important risk factors of HNSCC include cigarette smoking, alcohol consumption, or long-term use of betel nut chewing, and the most common site for intraoral carcinoma is the tongue. Oral tongue squamous cell carcinoma (OTSCC) is the largest form of tongue cancer, and recently, the incidence has been persistently increasing. Surgical resection is the gold standard for operable disease; in addition, chemotherapy, radiotherapy, targeted therapy, and immunotherapy or a combination of all these techniques can be considered as alternative options in clinical practice (Price & Cohen, 2012). Local recurrence and distant metastasis are the most critical factors that contribute to poor outcome and impaired quality of life (Camisasca et al., 2011; Lindenblatt Rde et al., 2012). Thus, identifying a potential biomarker in association with disease progression and prognosis in patients with OTSCC is a crucial research priority.

Jumonji domain-containing-3 (JMJD3), also called lysine-specific demethylase 6B (KDM6B), is a member of the family of jumonji C domain-containing histone demethylases and acts by modulating transcription through the removal of di- and trimethyl groups from histone H3 at lysine 27 (H3K27) (Agger et al., 2007; Hong et al., 2007). H3K27 trimethylation is associated with inactive gene promoters, whereas H3K27 monomethylation contributes to active gene promoters. Several studies have demonstrated the link between JMJD3 and tumor progression (Lagunas-Rangel, 2021; Sanchez et al., 2021). However, the role of JMJD3 in tumor suppression or enhancement of cancer cell proliferation remains controversial (Barradas et al., 2009; Xiang et al., 2007; Yin et al., 2019). Regarding tumor suppression, JMJD3 could inhibit tumor cell proliferation by regulating vitamin D or enhancing apoptosis through the nuclear translocation of FOXO1 in many cancer types, like colorectal cancer, non-small cell lung cancer or pancreatic cancer (Ma et al., 2015; Pereira et al., 2011; Tokunaga et al., 2016; Yamamoto et al., 2014). In contrast, JMJD3 could promote cancer cell invasion and tumor growth through epithelial–mesenchymal transition (EMT) and apoptosis through activating BCL2 or SLUG in various cancer types, such as gastric cancer, breast cancer, non-small cell lung cancer, hepatocellular carcinoma, ovarian cancer, renal cell carcinoma and multiple myeloma (Lee et al., 2021; Li et al., 2015; Liang et al., 2019; Ohguchi et al., 2017; Ramadoss, Chen & Wang, 2012; Shen et al., 2012; Svotelis et al., 2011; Tang et al., 2016; Xu et al., 2019; Zhang et al., 2019). Overexpression of JMJD3 results in cancer cell suppression via p16 and nuclear stabilization of p53 (Ene et al., 2012). The function of Rb is mediated by p16, and methylation of Rb facilitates the interaction with protein kinase CDK4; JMJD3/Rb interaction together links to heterochromatin, contributing to cancer senescence. On the other hand, JMJD3 is also involved in the activation of downstream pathway, such as p53 and p21, resulting in the induction of tumor growth arrest (Perrigue, Najbauer & Barciszewski, 2016).

However, the significance of JMJD3 in OTSCC remains unclear. We hypothesized that high expression of JMJD3, serving as a tumor suppressor gene via inducing p21 expression and regulating Rb pathway, is a novel mechanism contributing to the inhibition of tumor cell progression in OTSCC patients. The current study therefore aimed to investigate the crucial role of JMJD3 in the prognosis of OTSCC patients who underwent surgical resection.

## Materials and Methods

### Patient selection

Between January 2007 and December 2016, a total of 1,143 OTSCC patients treated at Kaohsiung Chang Gung Memorial Hospital were retrospectively reviewed. First, we excluded patients who had a history of any second primary malignancy or distant metastasis. Second, we also excluded those who received neoadjuvant chemotherapy, radiotherapy, or chemoradiotherapy. Third, only patients who underwent curative surgical resection were enrolled. Fourth, only patients with complete medical records were included. Finally, a total of 156 OTSCC patients were identified.

Tumor grade were classified as grade 1 (well differentiation), grade 2 (moderate differentiation) and grade 3 (poorly differentiation) by the pathologists based on how abnormal the tumor cells and the tumor tissue look under a microscope. The definition of smokers included “current smokers” who have smoked more than 100 cigarettes in their lifetime and has smoked in the last 28 days, and “ex-smokers” that means they have smoked more than 100 cigarettes in their lifetime but have not smoked in the last 28 days.

### Immunohistochemistry

The formalin fixed paraffin wax embedded OTSCC tissue was sectioned to be 4 µm in thickness for each patient. First, deparaffinization by incubation in a dry oven at 60 °C for 1 h, antigen retrieval in 10 mM citrate buffer (pH 6.0) in a hot water bath (95 °C) for 20 min, and peroxidase blocking with 0.3% hydrogen peroxide for 5 min. After that, the specimen was reacted with primary antibody against JMJD3 (Abcam, ab38113, 1:100, Cambridge, UK), and then with a ready-to-use visualization reagent consisting of goat secondary antibody. Then, tissue was incubated with polymer for 8 min and followed by 3–3′-diaminobenzidine for 10 min and hematoxylin for counterstaining. The slide of normal colon mucosa was used as a positive control; for the negative control, primary antibodies were omitted. The scores of these slides were investigated by 2 pathologists (WT Huang and SL Wang) blinded to clinicopathologic features or prognosis. The method to score the expression of JMJD3 was determined according to previous published study (Shen et al., 2012).

The proportions of JMJD3-expressing tumor cells were scored by immunoreactive score (IRS) system which is calculated by the product of the multiplying the staining intensity (no staining = 0, weak staining = 1, moderate staining = 2, strong staining = 3) and percentage of positive stained cells (0% = 0, 1–10% = 1, 11–50% = 2, 51–80% = 3, 81–100% = 4), resulting in IRS scores between 0 (no staining) and 12 (maximum staining) (Remmele & Stegner, 1987). A specimen with a sum score > 6 was regarded as high expression.

### Cell lines and culture

SAS and Cal 27, two cell lines of OTSCC, were purchased from American Type Culture Collection, and cultured in the Dulbecco’s modified Eagle’s medium, containing 10% of fetal bovine serum. Cells were cultured at 37 °C. We had performed the short tandem repeat profiling of these cell lines and the last date of authentication was 02 July 2020.

### Western blot analysis

GSK-J4, a UTX-specific pharmacological inhibitor (SML0701, Sigma-Aldrich, St. Louis, Missouri, USA), reconstituted in DMSO, with different concentrations (0, 2, 4, 8 µM) were given to these cells for 48 h. Whole-cell lysates of GSK-J4 treated cells were extracted using the fixture of RIPA lysis buffer (50 mM Tris, 150 mM NaCl, 1% NP40, 0.5% sodium deoxycholate, and 0.1% sodium dodecyl sulfate (SDS)) and subjected to western blot analyses. The membranes were incubated with primary antibodies against JMJD3 (ab169197, 1:2000, Abcam, Cambridge, MA, USA), Rb (#9309, 1:1000, cell signaling, Danvers, Massachusetts, USA), phosphorylated Rb (pRb) (ab173289, 1:1000, Abcam, Cambridge, UK), p21 (#2947, 1:1000, Cell Signaling, Danvers, Massachusetts, USA) and β-actin (A5441, 1:10000, Sigma-Aldrich, St. Louis, Missouri, USA) at 4 °C overnight. After that, the membranes were incubated with secondary antibodies for one hour at room temperature to reveal the immune detection. The protein signals were detected with Western Lightning Chemiluminescence Reagent Plus (Amersham Biosciences). All the experiments were repeated at least three times with similar results.

### Statistical analysis

Three independent experiments were performed and similar results were documented. The statistical analysis was performed using SPSS software (International Business Machines Corp, New York, USA). The Chi-square test was used to examine the difference of categorical variables. Disease-free survival (DFS) was calculated from the time from surgery to recurrence of tumor or death from any cause. Overall survival (OS) was defined as the duration between the time of diagnosis of OTSCC to death or last living contact. The Kaplan–Meier method was used for univariate analysis, and the log-rank test was performed to assess the difference. Multivariate analysis was examined using Cox proportional hazards model to identify the independent prognostic factors. A two-tailed *p* value of < 0.05 was considered to statistically significant.

### Ethics statement

The ethic approval for this study was obtained from the Chang Gung Medical Foundation Institutional Review Board (201901388B0). All procedures performed in studies were in accordance with the ethical standards of the institutional research committee and the World Medical Association Declaration of Helsinki. Written informed consent was waived by the Chang Gung Medical Foundation Institutional Review Board.

## Results

### Patient characteristics

We identified 156 OTSCC patients who underwent surgical resection at Kaohsiung Chang Gung Memorial Hospital between January 2007 and December 2016. All of the 156 OTSCC patients had an Eastern Cooperative Oncology Group performance status ≤1. In our study, 143 patients were male and 13 patients were female, and the median age was 53 years (range: 26–86 years). The study included 127 patients (81%) who had a history of cigarette smoking, 124 patients (80%) who had a history of alcohol consumption, and 116 patients (74%) who had a history of betel-nut chewing. Forty-five patients (29%) were diagnosed to have T1 status, 49 patients (32%) with T2 status, 13 patients (8%) with T3 status, and 49 patients (31%) with T4 status. Regarding the tumor N status, 86 patients (55%) had N0, 24 patients (15%) had N1, 43 patients (28%) had N2, and three patients (2%) had N3. There were 34 patients (22%) in stage I, 30 patients (19%) in stage II, 24 patients (15%) in stage III, 61 patients (39%) in stage IVA, and seven patients (5%) in stage IVB. Tumor grade analysis revealed that 91 patients (58%) were in grade 1, 59 patients (38%) were in grade 2, and six patients (4%) were in grade 3. Adjuvant radiotherapy or chemoradiotherapy were performed based on adverse risk factors, such as extracapsular extension, positive surgical margin, pathological T3 or T4 status, presentation of multiple lymph node involvement, lymphovascular or perineural invasion.

In the current study, the median follow-up period was 87.9 months for the 71 living survivors and 59.2 months (range: 1.0–112.1 months) for all 156 patients. The five-year DFS and OS rates were 46.2% and 50.0%, respectively. Detailed information is shown in Table 1.

**Table 1:** Characteristics of 156 patients with oral tongue squamous cell carcinoma receiving surgical resection.

Age (years)	53 (range: 26–86)	
Sex		
	male	143 (92%)
	female	13 (8%)
Pathological T status		
	T1	45 (29%)
	T2	49 (32%)
	T3	13 (8%)
	T4	49 (31%)
Pathological N status		
	N0	86 (55%)
	N1	24 (15%)
	N2	43 (28%)
	N3	3 (2%)
Pathological 8th AJCC Stage		
	I	34 (22%)
	II	30 (19%)
	III	24 (15%)
	IVA	61 (39%)
	IVB	7 (5%)
Histologic grade		
	1	91 (58%)
	2	59 (38%)
	3	6 (4%)
JMJD3 expression		
	Low expression	83 (53%)
	High expression	73 (47%)
Vascular invasion		
	Absent	131 (84%)
	Present	25 (16%)
Perineural invasion		
	Absent	87 (56%)
	Present	69 (44%)
Extracapsular extension		
	Absent	119 (76%)
	Present	37 (24%)
Margin status		
	Negative	145 (93%)
	Positive	11 (7%)
Smoking		
	Absent	29 (19%)
	Present	127 (81%)
Alcohol		
	Absent	32 (20%)
	Present	124 (80%)
Betel-nut chewing		
	Absent	40 (26%)
	Present	116 (74%)

**Notes.**

AJCC, American Joint Committee on Cancer.

### JMJD3 and clinical outcome

**Figure 1:**
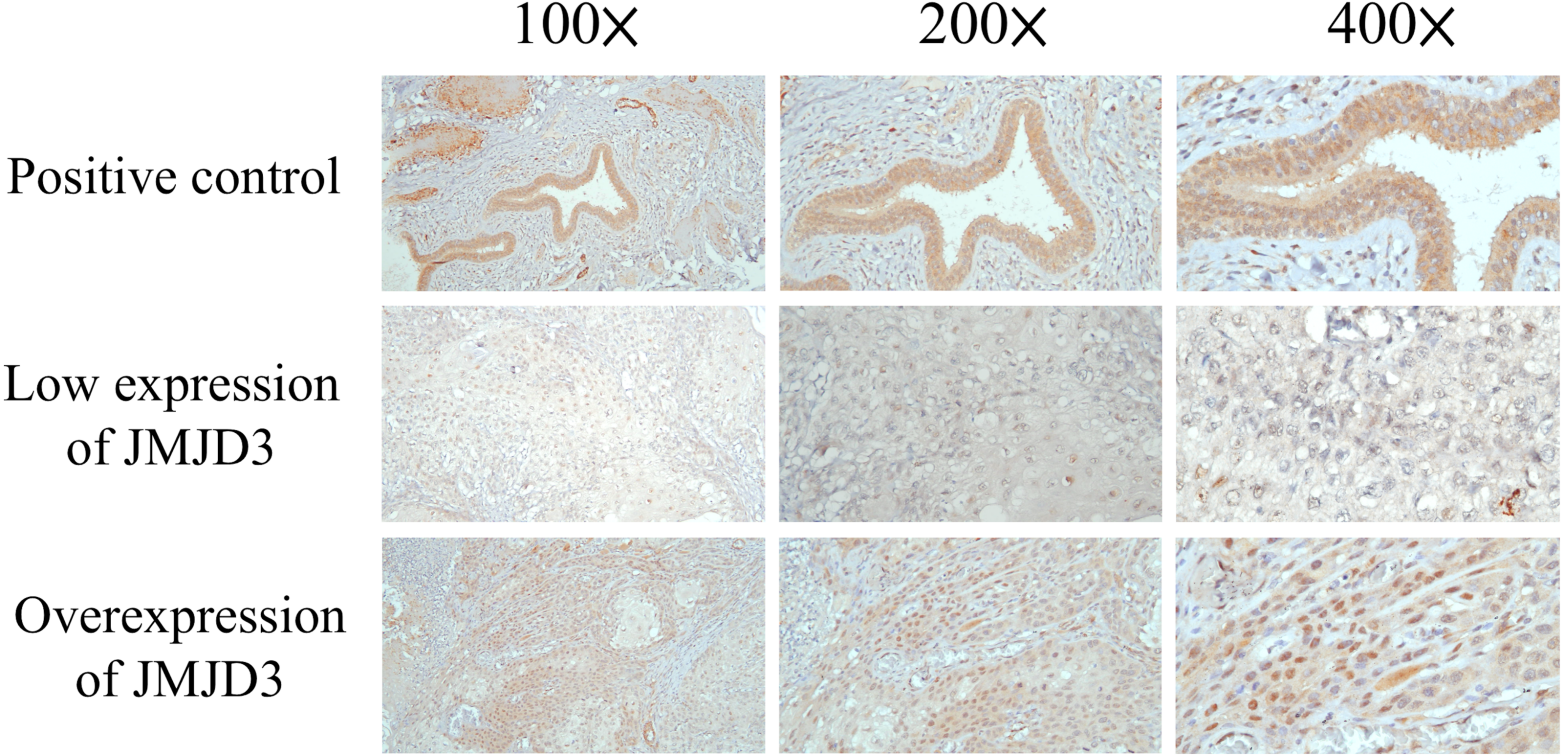
Immunohistochemistry. The immunohistochemical analysis of JMJD3 in oral tongue squamous cell carcinoma patients.

Results of the immunohistochemical staining of JMJD3 are shown in Fig. 1. Among the 156 patients, 73 patients (47%) had high expression of JMJD3 and 83 patients (53%) had low expression of JMJD3. The two groups were compared for baseline characteristics, and no significant difference was observed for age, sex, histologic grade, vascular invasion, surgical margin, alcohol consumption, cigarette smoking and betel-nut chewing. Patients with low expression of JMJD3 had a higher percentage of pathological T state, pathological N status, tumor stage, perineural invasion, and extracapsular extension than those with high expression of JMJD3. The comparison results are shown in Table 2.

**Figure 2:**
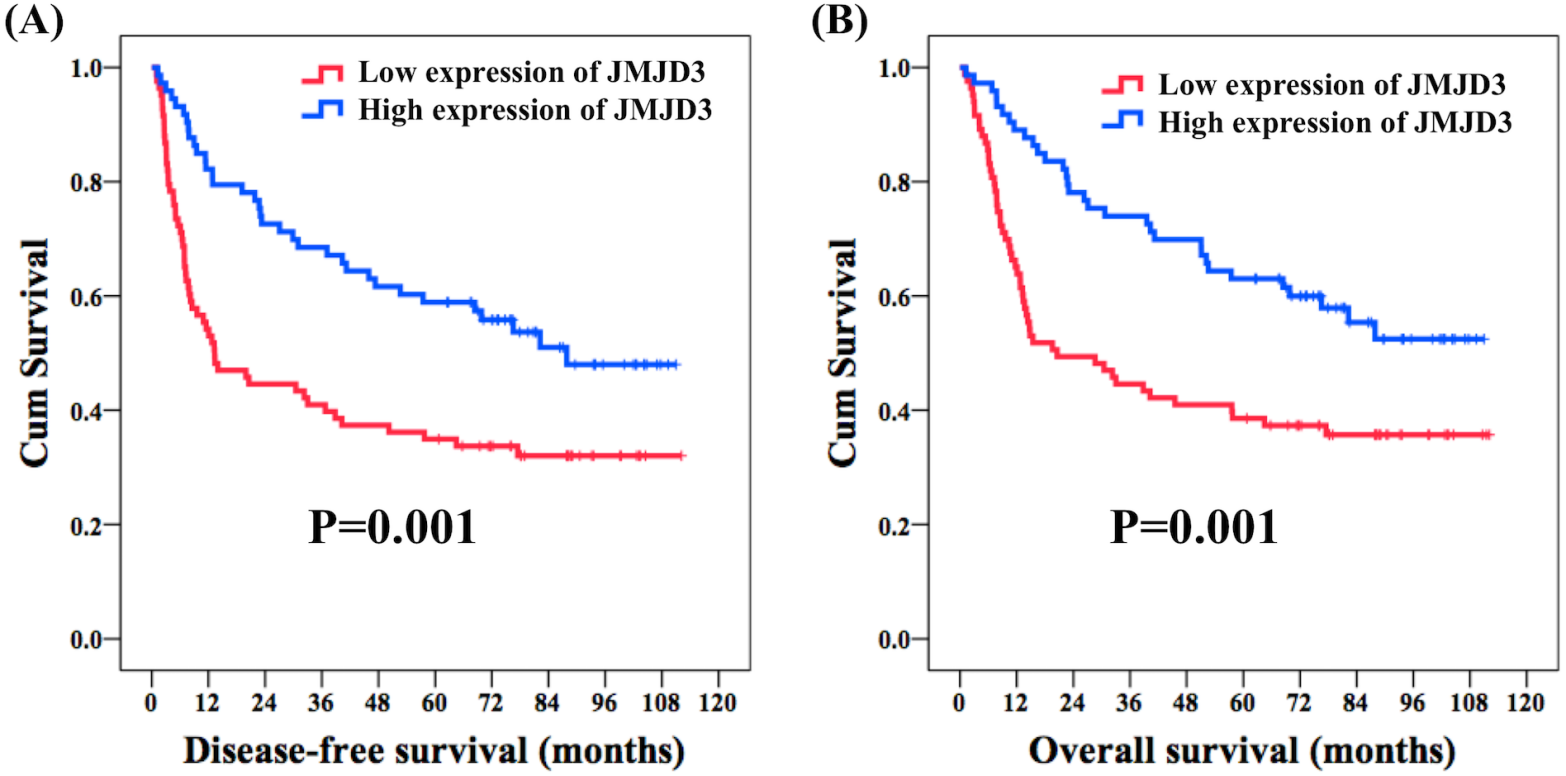
Survival. Kaplan–Meier curves in oral tongue squamous cell carcinoma patients according to expression of JMJD3. (A) Disease-free survival. (B) Overall survival.

With respect to DFS, age, sex, histologic grade, surgical margin, alcohol consumption, and betel-nut chewing did not meet statistical significance in the univariate analysis. The 62 patients with pathological T1-2 were found to have a better five-year DFS rate compared with the 94 patients with pathological T3-4 (53% versus 36%, *P* = 0.007); meanwhile, the 86 patients without nodal metastasis had a superior five-year DFS rate than the rest 70 patients with nodal metastasis (57% versus 33%, *P* < 0.001). A significant five-year DFS rate was mentioned in the 64 patients who had pathological stage I–II than in the 92 patients who had pathological stage III–IV (59% versus 37%, *P* = 0.001). One hundred thirty-one patients without vascular invasion were found to have a superior five-year DFS rate compared to 25 patients with vascular invasion (50% versus 28%, *P* = 0.028), and a better five-year DFS rate was observed in 87 patients without perineural invasion than in the other 69 patients with perineural invasion (55% versus 35%, *P* = 0.003). A significantly improved five-year DFS rate was found in 119 patients without extracapsular extension compared to that in 37 patients with extracapsular extension (52% versus 27%, *P* < 0.001); in addition, 29 patients without cigarettes smoking had a better five-year DFS rate in comparison with 127 smokers (66% versus 42%, *P* = 0.028). Seventy-three patients who had high expression of JMJD3 had a better five-year DFS rate than the rest 83 patients with a low expression of JMJD3 (59% versus 35%, *P* = 0.001, Fig. 2A). Multivariate analysis showed that pathological T1-2 status (*P* = 0.038, hazard ratio (HR): 0.63, 95% confidence interval (CI) [0.40–0.97]), grade 1 (*P* = 0.012, HR: 0.58, 95% CI [0.38–0.88]), no extracapsular extension (*P* = 0.002, HR: 0.48, 95% CI [0.30–0.76]), and high expression of JMJD3 (*P* = 0.011, HR: 0.57, 95% CI [0.37–0.88]) were the independent prognostic factors of a superior five-year DFS rate.

**Table 2:** Associations between JMJD3 expression and clinicopathological parameters in 156 patients with oral tongue squamous cell carcinoma receiving surgical resection.

Parameters		JMJD3 expression		
		Low expression (*N* = 83)	High expression (*N* = 73)	*P* value
Age				
	<53y/o	40 (48%)	36 (49%)	0.89
	≥53y/o	43 (52%)	37 (51%)	
Sex				
	male	76 (92%)	67 (92%)	0.96
	female	7 (8%)	6 (8%)	
Pathological T status				
	T1 + T2	41 (49%)	53 (73%)	0.003^*^
	T3 + T4	42 (51%)	20 (27%)	
Pathological N status				
	Nodal negative	33 (40%)	53 (73%)	<0.001^*^
	Node positive	50 (60%)	20 (27%)	
Pathological 8th AJCC Stage				
	I + II	21 (25%)	43 (59%)	<0.001^*^
	III + IV	62 (75%)	30 (41%)	
Histologic grade				
	1	47 (57%)	44 (60%)	0.65
	2+3	36 (43%)	29 (40%)	
Vascular invasion				
	Absent	69 (83%)	62 (85%)	0.76
	Present	14 (17%)	11 (15%)	
Perineural invasion				
	Absent	40 (48%)	47 (64%)	0.042^*^
	Present	43 (52%)	26 (36%)	
Extracapsular extension				
	Absent	57 (69%)	62 (85%)	0.017^*^
	Present	26 (31%)	11 (15%)	
Margin status				
	Negative	76 (92%)	69 (95%)	0.47
	Positive	7 (8%)	4 (5%)	
Smoking history				
	Absent	14 (17%)	15 (21%)	0.56
	Present	69 (83%)	58 (79%)	
Alcohol history				
	Absent	15 (18%)	17 (23%)	0.42
	Present	68 (82%)	56 (77%)	
Betel-nut chewing history				
	Absent	21 (25%)	19 (26%)	0.92
	Present	62 (75%)	54 (74%)	

**Notes.**

AJCC, American Joint Committee on Cancer.

* Statistically significant.

In the analysis of OS, the univariate analysis revealed that there were no significant differences in age, sex, histologic grade, alcohol consumption, and betel nut chewing. The 62 patients who had pathological T1-2 were found to have a better five-year OS rate compared with the 94 patients who had pathological T3-4 (59% versus 37%, *P* = 0.002); meanwhile, the 86 patients without nodal metastasis had a superior five-year OS rate than the rest 70 patients with nodal metastasis (61% versus 37%, *P* < 0.001). The 64 patients who had pathological stage I–II were found to have a significantly better five-year OS rate than the 92 patients who had pathological stage III–IV (63% versus 41%, *P* = 0.001). The 131 patients without vascular invasion were mentioned to have a better five-year OS rate than the 25 patients with vascular invasion (54% versus 28%, *P* = 0.028), and a better five-year OS rate was observed in the 87 patients without perineural invasion than in the other 69 patients with perineural invasion (58% versus 41%, *P* = 0.012). The 119 patients without extracapsular extension had a significantly superior five-year OS rate compared to the 37 patients with extracapsular extension (56% versus 30%, *P* < 0.001); meanwhile, a superior five-year OS rate was observed for the 145 patients without surgical margin compared with the 11 patients with a surgical margin (52% versus 27%, *P* = 0.016). The 29 patients without cigarettes smoking had a superior five-year OS rate compared to the 127 smokers (72% versus 45%, *P* = 0.016). The 73 patients who had high expression of JMJD3 had a better five-year OS rate than the other 83 patients with low expression of JMJD3 (63% versus 39%, *P* = 0.001, Fig. 2B). In the multivariate analysis, pathological T1-2 status (*P* = 0.030, HR: 0.60, 95% CI [0.37–0.95]), grade 1 (*P* = 0.007, HR: 0.54, 95% CI [0.34–0.85]), vascular invasion (*P* = 0.034, HR: 0.55, 95% CI [0.32–0.95]), no extracapsular extension (*P* = 0.003, HR: 0.48, 95% CI [0.30–0.78]), and high expression of JMJD3 (*P* = 0.013, HR: 0.57, 95% CI [0.36–0.88]) were the independent prognostic factors of a better five-year OS rate. Survival outcome results of the univariate and multivariable analyses are shown in Tables 3 and 4, respectively.

**Table 3:** Disease-free survival. Results of univariate and multivariable analyses of prognostic factors for disease-free survival (DFS) in 156 patients with oral tongue squamous cell carcinoma receiving surgical resection.

Parameters		Number of patients	Univariate analysis		Multivariable analysis	
			5-year DFS (%)	*P* value	HR (95% CI)	*P* value
Age						
	<53y/o	76 (49%)	51%	0.15		
	≥53y/o	80 (51%)	41%			
Sex						
	male	143 (92%)	45%	0.19		
	female	13 (8%)	62%			
Pathological T status						
	T1 + T2	94 (60%)	53%	0.007^*^	0.63 (0.40–0.97)	0.038^*^
	T3 + T4	62 (40%)	36%			
Pathological N status						
	Nodal negative	86 (55%)	57%	<0.001^*^		
	Nodal positive	70 (45%)	33%			
Pathological 8th AJCC Stage						
	I + II	64 (41%)	59%	0.001^*^		
	III + IV	92 (59%)	37%			
Histologic grade						
	1	91 (58%)	52%	0.057	0.58 (0.38–0.88)	0.012^*^
	2+3	65 (42%)	39%			
Vascular invasion						
	Absent	131 (84%)	50%	0.028^*^		
	Present	25 (16%)	28%			
Perineural invasion						
	Absent	87 (56%)	55%	0.003^*^		
	Present	69 (44%)	35%			
Extracapsular extension						
	Absent	119 (76%)	52%	<0.001^*^	0.48 (0.30–0.76)	0.002^*^
	Present	37 (24%)	27%			
Margin status						
	Negative	145 (93%)	48%	0.058		
	Positive	11 (7%)	27%			
Smoking history						
	Absent	29 (19%)	66%	0.028^*^		
	Present	127 (81%)	42%			
Alcohol history						
	Absent	32 (21%)	50%	0.41		
	Present	124 (79%)	45%			
Betel-nut chewing history						
	Absent	40 (26%)	53%	0.19		
	Present	116 (74%)	44%			
JMJD3 expression						
	Low expression	83 (53%)	35%	0.001^*^		
	High expression	73 (47%)	59%		0.57 (0.37–0.88)	0.011^*^

**Notes.**

AJCC American Joint Committee on Cancer HR hazard ratio CI confidence interval

* Statistically significant.

**Table 4:** Overall survival. Results of univariate and multivariable analyses of prognostic factors for overall survival (OS) in 156 patients with oral tongue squamous cell carcinoma receiving surgical resection.

Parameters		Number of patients	Univariate analysis		Multivariable analysis	
			5-year OS (%)	*P* value	HR (95% CI)	*P* value
Age						
	<53y/o	76 (49%)	54%	0.25		
	≥53y/o	80 (51%)	46%			
Sex						
	male	143 (92%)	48%	0.11		
	female	13 (8%)	69%			
Pathological T status						
	T1 + T2	94 (60%)	59%	0.002^*^	0.60 (0.37–0.95)	0.030^*^
	T3 + T4	62 (40%)	37%			
Pathological N status						
	Nodal negative	86 (55%)	61%	<0.001^*^		
	Nodal positive	70 (45%)	37%			
Pathological 8th AJCC Stage						
	I + II	64 (41%)	63%	0.001^*^		
	III + IV	92 (59%)	41%			
Histologic grade						
	1	91 (58%)	55%	0.079	0.54 (0.34–0.85)	0.007^*^
	2+3	65 (42%)	43%			
Vascular invasion						
	Absent	131 (84%)	54%	0.028^*^	0.55 (0.32–0.95)	0.034^*^
	Present	25 (16%)	28%			
Perineural invasion						
	Absent	87 (56%)	58%	0.012^*^		
	Present	69 (44%)	41%			
Extracapsular extension						
	Absent	119 (76%)	56%	<0.001^*^	0.48 (0.30–0.78)	0.003^*^
	Present	37 (24%)	30%			
Margin status						
	Negative	145 (93%)	52%	0.016^*^		
	Positive	11 (7%)	27%			
Smoking history						
	Absent	29 (19%)	72%	0.016^*^		
	Present	127 (81%)	45%			
Alcohol history						
	Absent	32 (21%)	56%	0.32		
	Present	124 (79%)	48%			
Betel-nut chewing history						
	Absent	40 (26%)	60%	0.079		
	Present	116 (74%)	47%			
JMJD3 expression						
	Low expression	83 (53%)	39%	0.001^*^		
	High expression	73 (47%)	63%		0.57 (0.36–0.88)	0.013^*^

**Notes.**

AJCC American Joint Committee on Cancer HR hazard ratio CI confidence interval

* Statistically significant.

### Inhibition of JMJD3 by GSK-J4

**Figure 3:**
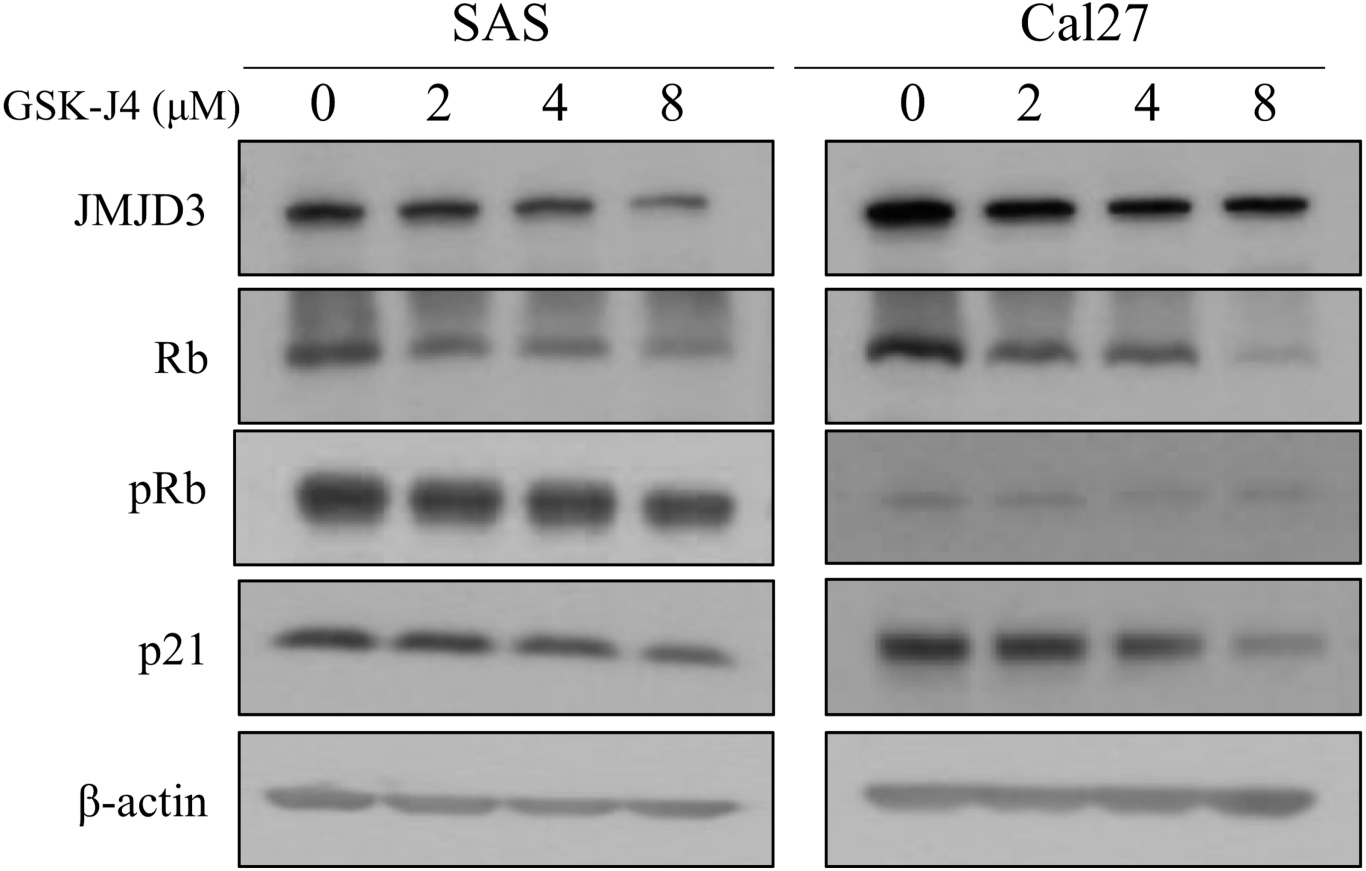
Western blot analysis. Western blot analysis of JMJD3 expression and the downstream signaling pathway in the SAS and Cal 27 cell lines. The protein expression profiles of JMJD3, p21, Rb and pRb were examined in the presence or absence of GSK-J4 treatment in the oral tongue squamous cell carcinoma cells by Western blotting.

In this *in vitro* study, western blot analyses were done to determine the expression of JMJD3, Rb, and p21 in the OTSCC cell lines which were treated with different concentrations of GSK-J4. Rb is a well-known tumor suppressor gene and p21 is a potent cyclin-dependent kinase inhibitor (Kent & Leone, 2019; Sherr & McCormick, 2002). Our data demonstrated that the expression of JMJD3 was decreased in GSK-J4-treated cell lines compared to that in the control cells; in addition, the expression of Rb, pRb and p21 were inhibited. The results are shown in Fig. 3.

## Discussion

**Figure 4:**
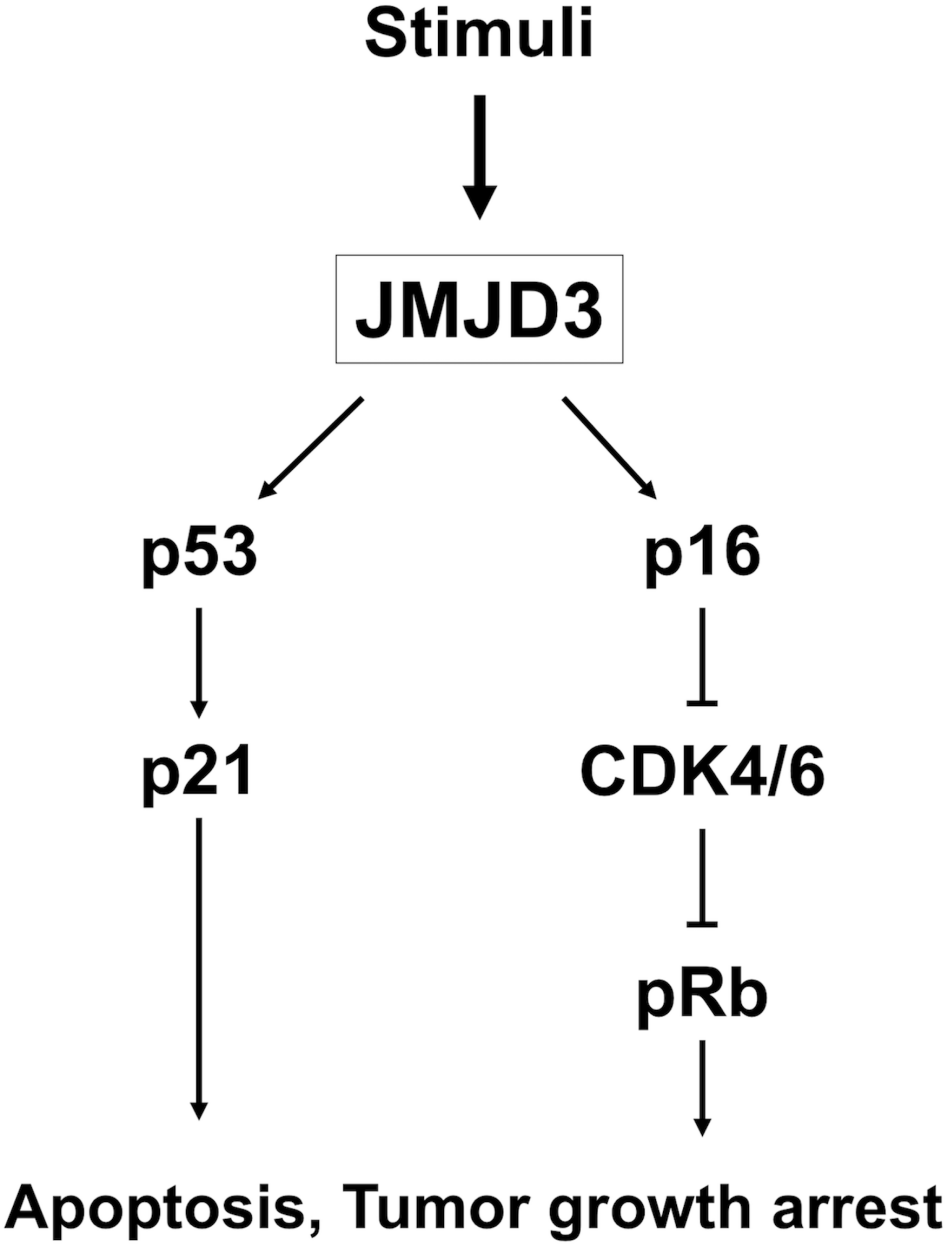
The mechanism of JMJD3. The mechanism of JMJD3 in the tumor cell arrest.

Histone demethylases are epigenetic regulators and play an important role in cancer as oncogenes and tumor suppressor genes. Increased H3K27me3 expression is reported to be associated with dysregulation of gene expression in various malignancies, and the level of H3K27me3 is regulated by the histone demethylase JMJD3 (Daures et al., 2018; Kondo et al., 2008; Ngollo et al., 2014a; Ngollo et al., 2014b). JMJD3 catalyzes the transition of H3K27me3 to H3K27me2 or H3K27me1 from a repressive to an active chromatin conformation (Zhang et al., 2015). JMJD3 is involved in carcinogenesis, such as proliferation, differentiation, apoptosis, and aging through many signaling pathways, and the expression pattern of JMJD3 in different cancers is still controversial (Lagunas-Rangel, 2021; Park et al., 2016; Pereira et al., 2011; Tokunaga et al., 2016; Xiang et al., 2007; Zhang et al., 2020). Growing evidence have demonstrated that JMJD3 induces p16 and p21 activation via up-regulation of P14, Rb and p53, contributing to cell cycle arrest and inhibition of tumor proliferation; this means JMJD3 may serve as a tumor suppressor gene, and the mechanism of JMJD3 in the tumor cell arrest in our study was shown in Fig. 4 (Ene et al., 2012; Zhao et al., 2015; Zhao et al., 2013). On the other hand, the prognostic value of JMJD3 was also validated in other independent human cancer cohorts, like TCGA database. In renal clear cell carcinoma cohort from TCGA database, patients with high expression of JMJD3 had superior OS compared to those with low expression of JMJD3 (*P* = 0.00071) (Chandrashekar et al., 2017). In pancreatic adenocarcinoma cohort from TCGA database, a borderline better OS was mentioned to be in patients with high expression of JMJD3 than those with low expression of JMJD3 (*P* = 0.11) (Chandrashekar et al., 2017).

In our study, univariate and multivariable analyses showed that low pathological T status, low tumor grade, no extracapsular extension and JMJD3 high expression were better prognostic factors of DFS and OS. There is controversy regarding whether JMJD3 acts as a tumor suppressor or oncoprotein. Pereira et al. (2011) reported that JMJD3 could suppress tumor proliferation through the regulation of vitamin D in colon cancer. Silencing JMJD3 promoted EMT inducers and mesenchymal markers and inhibited epithelial proteins. In another colorectal cancer study, inhibition of JMJD3 significantly induced cancer cell growth through cell cycle progression and apoptosis suppression, and low expression of JMJD3 was an independent predictor of poor outcome (Tokunaga et al., 2016). Yamamoto et al. (2014) reported that JMJD3 mediates oncogenic KRAS-induced senescence in pancreatic cancer. The high expression of JMJD3 decreased the malignant grade progression, and inhibition of JMJD3 enhanced tumor sphere formation, peritoneal dissemination and liver metastasis as revealed through *in vivo* studies. In our study, high expression of JMJD3 was found to be associated with better five-year DFS and OS rates, suggesting the role of JMJD3 as a tumor suppressor in OTSCC.

The Rb gene is one of the most frequently mutated genes in tumors and well-known to act as a tumor suppressor gene (Kent & Leone, 2019; Sherr & McCormick, 2002). The dysfunction of the Rb gene are involved in several aggressive phenotypes, such as proliferation, invasion, metastasis and drug resistance. Zhao et al. (2015) reported that JMJD3 can demethylate the non-histone protein Rb and high expression of JMJD3 enhanced cellular senescence. In addition, the interaction of Rb and protein kinase CDK4 was impeded by JMJD3-mediated demethylation of Rb, resulting in decreased level of phosphorylation of Rb (Zhao et al., 2015). Another study reported by Zhao revealed that JMJD3 is a potent negative regulator of reprogramming, and inhibits reprogramming by upregulating expression of p21 (Zhao et al., 2013). On the contrary, Tian et al. (2012) reported that JMJD3 promoted the tumor cell proliferation and enhanced the progress of cell cycle through the inhibition of p21 expression in lung cancer cells. Therefore, the role of JMJD3 may be controversial in several cancer type. In the current study, the expression of JMJD3 was inhibited by GSK-J4, resulting in reduced level of Rb and p21. This mechanism may explain the role of JMJD3 in the prognosis of OTSCC.

There are several limitations in our study. First, this was a retrospective analysis at a single institution, and the relatively small number of patients may limit this study’s statistical power. Second, neither the comprehensive mechanisms of JMJD3 and downstream pathways were investigated nor it was explored how JMJD3 mediates tumor cell growth, invasion, and metastasis. Nonetheless, this study is unique in that this is the largest series of OTSCC patients who received surgical resection and may provide powerful findings to understand the prognostic role of JMJD3 in OTSCC.

## Conclusion

Our study confirmed that high expression of JMJD3 is a good prognostic factor in OTSCC patients who underwent surgical resection. Further research in a large population is needed to confirm our findings and clarify the complex mechanism of JMJD3 in OTSCC.

## Supplemental Information

10.7717/peerj.13759/supp-1 Supplemental Information 1 Raw data

10.7717/peerj.13759/supp-2 Supplemental Information 2 Short tandem repeat profiles

10.7717/peerj.13759/supp-3 Supplemental Information 3 Protein expression on the western blotting

## Additional Information and Declarations

### Data Availability

The following information was supplied regarding data availability: The raw data is available in the Supplementary File.
