# Supplementary material for: JMJD3 suppresses tumor progression in oral tongue squamous cell carcinoma patients receiving surgical resection
Source: PeerJ. 2022 Jul 13;10:e13759. doi: 10.7717/peerj.13759 (PMC9288160; doi:10.7717/peerj.13759)

Cell Line DNA Typing Report

Case Number: CID20200042

Report Date: 07/08/2020

Genelabs Life Science Corp.
12F.-6, No.3, Yuanqu St.,
Nangang Dist.,
Taipei City 115, Taiwan
TEL: +886-2-26557678
FAX: +886-2-26557572
E-mail: cellid@genelabs.com.tw

Sample Information:

i. Applicant Name: 邱泰然 Tai-Jan Chiu

ii. Institution: 高雄長庚紀念醫院血液腫瘤科 Department of Hematology-Oncology, Kaohsiung Chang Gung Memorial Hospital

iii. Sample Description: SAS

iv. Sample type: Cell Pellet

v. Sample Received Date: 07/02/2020

Test Description:

DNA of the sample is extracted by Roche MagNA Pure Compact System.

DNA conc. = 143.3 ng/µl; OD260/280 = 2.00 ; OD260/230 = 2.26

The STR loci are amplified by Promega GenePrint® 24 System.

The CE analysis is performed on ABI PRISM 3730 GENETIC ANALYZER.

The raw data is analyzed by GeneMapper® Software V3.7.

The STR analysis is operated and reported by Mission Biotech.

This report is issued by:

Laboratory Director　　　General Manager

STR Analysis Result:

ANSI/ATCC ASN-0002 STR Loci	Repeat Numbers	Extended STR Loci	Repeat Numbers	Extended STR Loci	Repeat Numbers
D5S818	9,9	D3S1358	16,17	DYS391*	N/A
D13S317	10,12	D1S1656	11,15	D8S1179	11,13
D7S820	11,12	D2S441	11,11	D12S391	18,18
D16S539	12,13	D10S1248	14,14	D19S433	14,14.2
vWA	14,17	Penta E	5,20	FGA	24,24
TH01	6,7	D18S51	15,15	D22S1045	16,16
Amelogenin	X	D2S1338	20,24	Case Number: CID20200042<br>Test Date: 07/06/2020	
TPOX	11,12	Penta D	10,13		
CSF1PO	11,11	D21S11	30,30		

Human Cell Line DNA Typing Report

Allele Report

Case Number: CID20200042

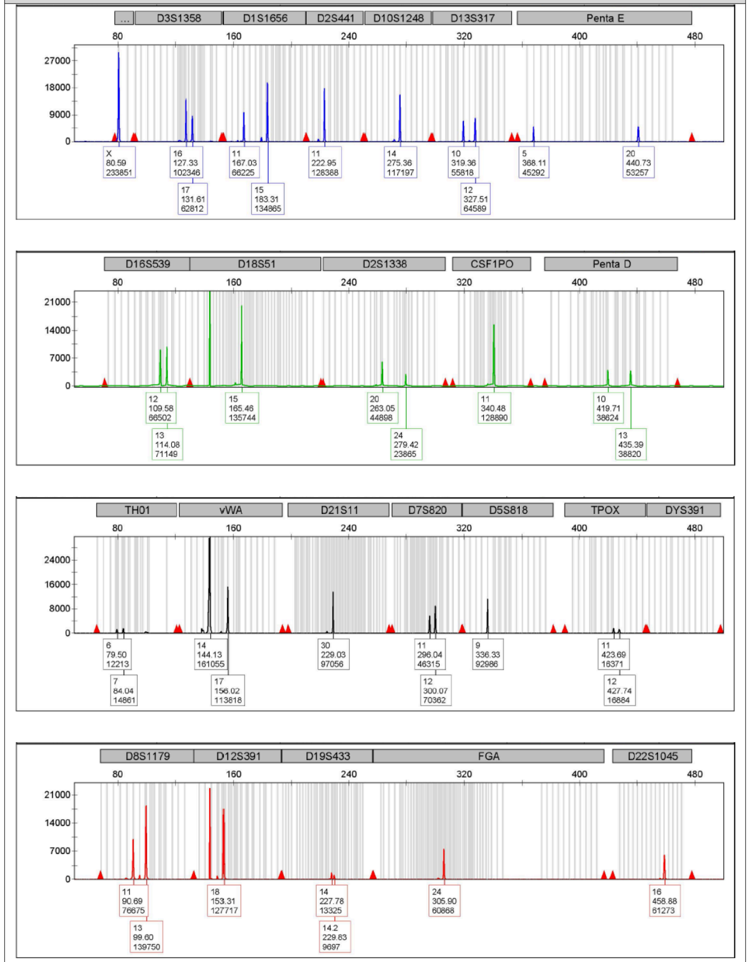

Cell Line DNA Typing Report

Case Number: CID20200040

Report Date: 07/08/2020

Genelabs Life Science Corp.
12F.-6, No.3, Yuanqu St.,
Nangang Dist.,
Taipei City 115, Taiwan
TEL: +886-2-26557678
FAX: +886-2-26557572
E-mail: cellid@genelabs.com.tw

Sample Information:

i. Applicant Name: 邱泰然 Tai-Jan Chiu

ii. Institution: 高雄長庚紀念醫院血液腫瘤科 Department of Hematology-Oncology, Kaohsiung Chang Gung Memorial Hospital

iii. Sample Description: CAL27

iv. Sample type: Cell Pellet

v. Sample Received Date: 07/02/2020

Test Description:

DNA of the sample is extracted by Roche MagNA Pure Compact System.

DNA conc. = 113.0 ng/μl; OD260/280 = 2.00; OD260/230 = 2.26

The STR loci are amplified by Promega GenePrint® 24 System.

The CE analysis is performed on ABI PRISM 3730 GENETIC ANALYZER.

The raw data is analyzed by GeneMapper® Software V3.7.

The STR analysis is operated and reported by Mission Biotech.

This report is issued by:

Laboratory Director   General Manager

STR Analysis Result:

ANSI/ATCC ASN-0002 STR Loci	Repeat Numbers	Extended STR Loci	Repeat Numbers	Extended STR Loci	Repeat Numbers
D5S818	11,12	D3S1358	16,16	DYS391*	N/A
D13S317	10,11	D1S1656	13,16	D8S1179	13,15
D7S820	10,10	D2S441	10,11.3	D12S391	18.3,20
D16S539	11,12	D10S1248	12,16	D19S433	14,15.2
vWA	14,17	Penta E	7,7	FGA	25,25
TH01	6,9.3	D18S51	13,13	D22S1045	13,16
Amelogenin	X	D2S1338	23,24	Case Number: CID20200040<br>Test Date: 07/06/2020	
TPOX	8,8	Penta D	9,10		
CSF1PO	10,12	D21S11	28,29		

Human Cell Line DNA Typing Report

Allele Report

Case Number: CID20200040

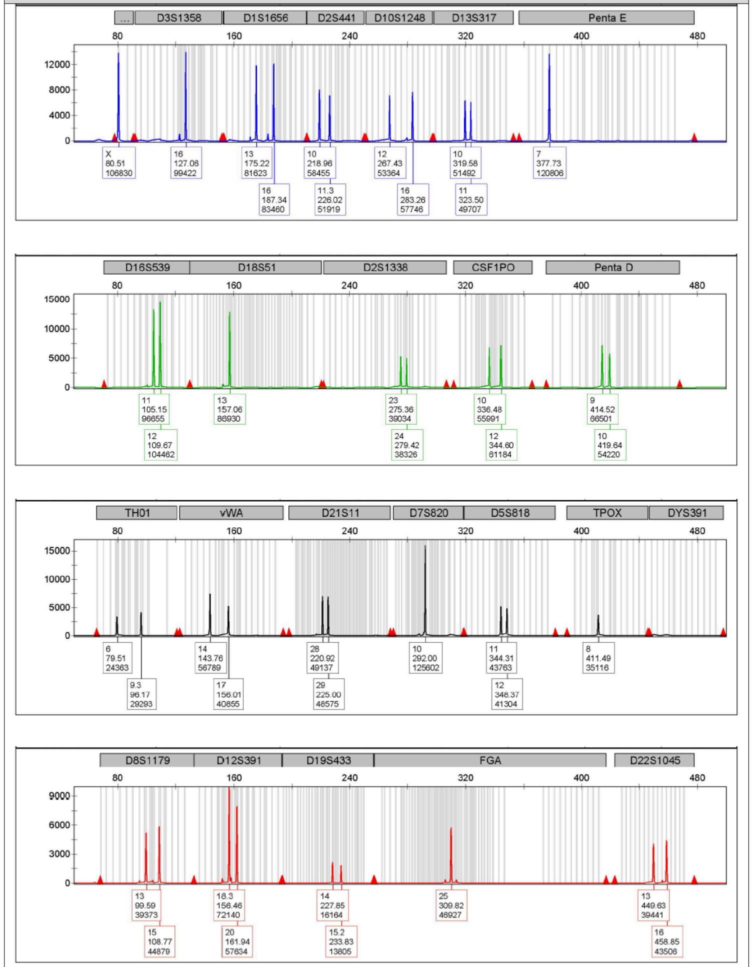

Supplement: Supplemental Information 2 [file peerj-10-13759-s002.pdf]
