# Supplementary material for: JMJD3 suppresses tumor progression in oral tongue squamous cell carcinoma patients receiving surgical resection
Source: PeerJ. 2022 Jul 13;10:e13759. doi: 10.7717/peerj.13759 (PMC9288160; doi:10.7717/peerj.13759)

2021.04.09 HNCC + GSK-J4 (JMJD3/UTX inhibitor)

SAS Cal27

GSK-J4 (uM) 0 2 4 8 0 2 4 8

JMJD3 (1:2000)

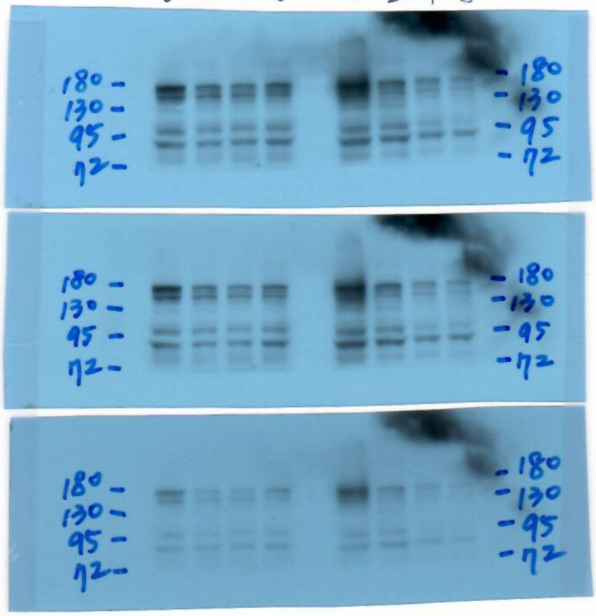

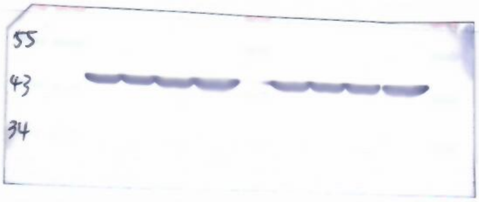

Actin

UTX (1:2000)

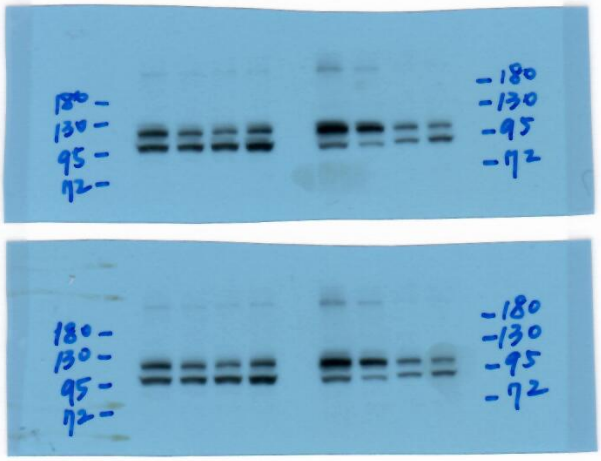

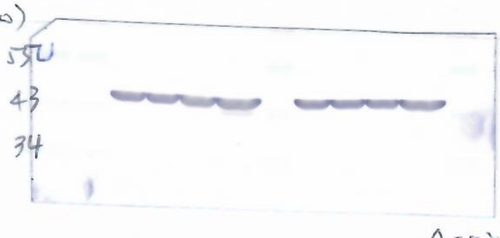

Actin

8% SDS-PAGE

lysate: 50 ug

Running condition: 80-130V

Transfer condition: 90 min

Blocking : 5% Skim-milk RT 1hr

1°Ab : 4°C O/N ~19hr

1°Ab wash: 90 rpm / 15min x4

2°Ab : 1 hr (RT)

2°Ab wash: 90 rpm / 10min x4

2020.08.28

HNCC + GSK-J4 (JMJD3/UTX inhibitor)

SAS Cal27

GSK-J4 (uM) 0 2 4 8 0 2 4 8 JMJD3

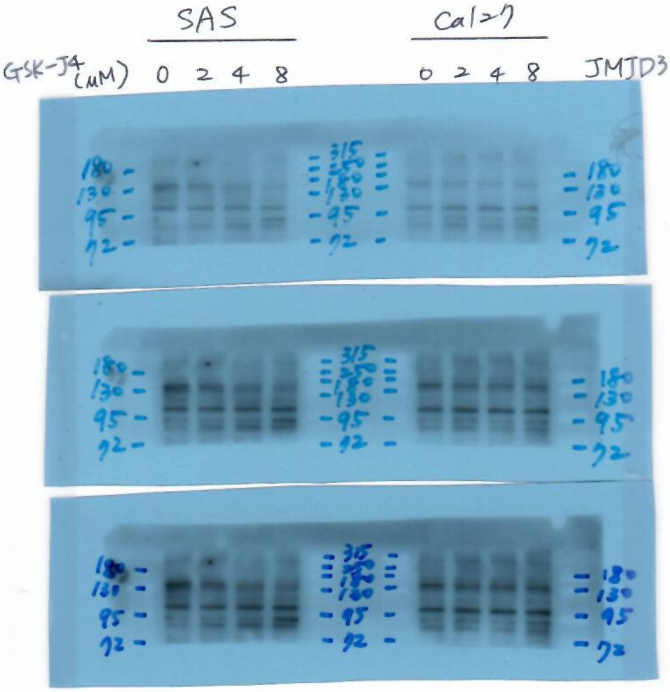

SAS Cal27

GSK-J4 (uM) 0 2 4 8 0 2 4 8 UTX

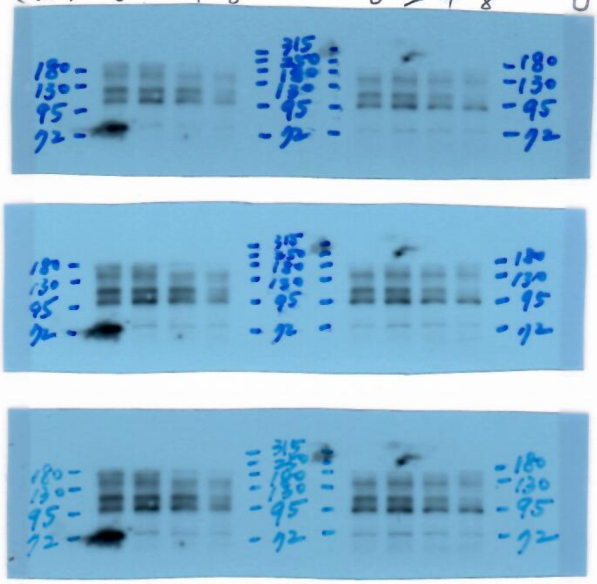

p21

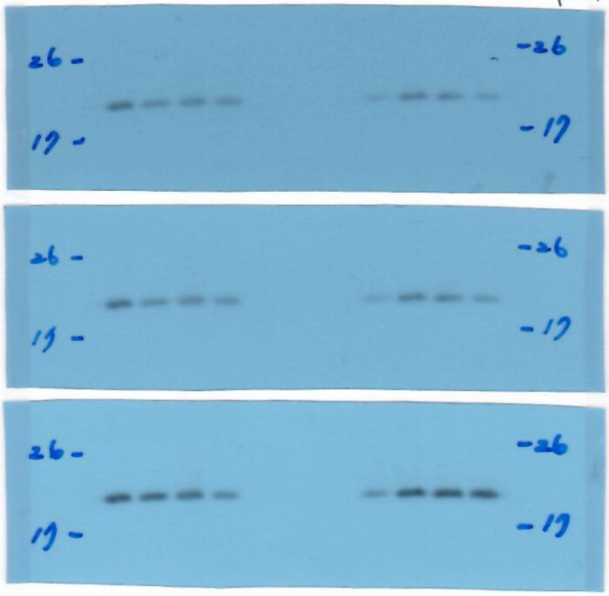

Rb

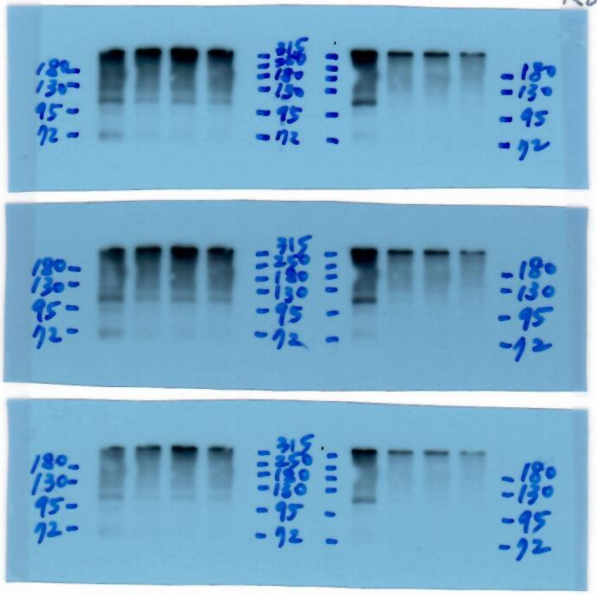

Supplement: Supplemental Information 3 [file peerj-10-13759-s003.zip › Oridinal data 20210817.pdf]
